# Long term sexual outcomes of Mayer Rokitansky Küster Hauser Syndrome patients after Uncu-modified Davydov procedure

**DOI:** 10.52054/FVVO.15.3.091

**Published:** 2023-09-24

**Authors:** K Aslan, T.B. Gurbuz, A Orhan, I Kasapoglu, K Ozerkan, G Uncu

**Affiliations:** Bursa Uludag University School of Medicine Department of Obstetrics and Gynecology, Minimally Invasive Surgery Unit, Gorukle, Bursa, Turkey

**Keywords:** Mullerian Agenesis, Laparoscopy, Neovaginoplasty, Mayer Rokitansky Küster Hauser Syndrome

## Abstract

**Background:**

Mayer-Rokitansky-Küster-Hauser (MRKH) syndrome has an incidence of 1 in 4000. The absence of the vagina and uterus results in sexual dysfunction and infertility. The first-line treatment is vaginal dilatation. There exists a number of second-line surgical options including the Uncu-modified Davydov procedure.

**Objective:**

To determine the complication rate, anatomical outcomes, and long-term sexual outcomes of MRKH syndrome patients after Uncu-modified Davydov procedure.

**Materials and Methods:**

Patients with MRKH syndrome who underwent paramesonephric remnant-supported laparoscopic double-layer peritoneal pull-down vaginoplasty (aka Uncu-modified Davydov procedure) between January 2008 and December 2021. The procedure involves laparoscopic circular dissection of the pelvic peritoneum followed by pulling down, through the opened vaginal orifice, and suturing the vaginal cuff with the support of uterine remnants. The long-term complication rate, anatomical outcomes, and sexual function outcomes (as measured by Female Sexual Function Index (FSFI)) were ascertained.

**Main Outcome Measures:**

The long-term complication rate, anatomical outcomes and FSFI survey results.

**Results:**

A total of 50 patients with MRKH syndrome underwent the Uncu-modified Davydov procedure between Jan 2008- Dec 2021. There were four perioperative complications: three bladder injuries (6%) and one rectal serosa injury (2%). Four long-term postoperative complications were identified: one vesicovaginal fistula (2%), one recto-vaginal fistula (2%), and two vaginal stenoses (4%). All patients were physically examined at least one year after surgery. The mean vaginal length was 8.4 + 1.9 cm. The mean FSFI score was 31.5 + 3.9 (minimum score of 24, maximum score of 36).

**Conclusion:**

The Uncu-modified Davydov procedure has been demonstrated to be a safe and effective treatment option with high female sexual function index scores for patients with MRKH syndrome.

**What is new?:**

The long-term complication rate, anatomical and sexual outcomes of Uncu-modified laparoscopic peritoneal pull-down vaginoplasty were reported in this study. The results indicated that the surgical approach could be used in selective MRKH patients who failed first-line self-dilatation therapy.

## Introduction

Since Mayer-Rokitansky-Küster-Hauser syndrome was first described (Pizzo et al., 2013), numerous vaginoplasty methods have been invented. ACOG (American College of Obstetrics and Gynecology) advises vaginal non-surgical, self-dilatation method as first-line treatment in MRKH patients (Committee on Adolescent Health Care, 2018). When self- dilatation is ineffective or declined (because of personal, cultural, or religious reasons) the second line treatment is the surgical approach: neovagina creation.

There are three principal surgical methods for neovagina creation. The first approach is the McIndoe method (Banister and McIndoe, 1938), a vaginal approach consisting of insertion a created vagina (with numerous materials like an autologous skin graft, in-vitro cultured vaginal tissue, acellular collagen, amniotic membrane, fish skin or buccal mucosa) into the dissected pouch between rectum and bladder (Teng et al., 2019; Sabatucci et al., 2019; Motta et al., 2017; Dias et al., 2020; Vatsa et al., 2017; Wu et al., 2020; Jackson and Rosenblatt, 1994; Rothman, 1972). The second approach is the Vecchietti operation which requires abdomen-vaginal traction with special instruments (Vecchietti, 1965; Cezar et al., 2014; Yang et al., 2021). This method includes both vaginal and abdominal (laparotomy or laparoscopy) surgery. The last method is Davydov vaginoplasty. In this technique, an artificial canal is created between the blind vaginal introitus and the peritoneal cavity by dissection. The parietal peritoneum is pulled down by the vaginal approach, and the vaginal cuff is closed with purse-string sutures (Davydov and Zhvitiashvili, 1974). The Uncu-modified Davydov procedure entails paramesonephric remnant- supported laparoscopic double-layer peritoneal pull- down vaginoplasty - which we previously described (Uncu et al., 2018). It is entailing an entirely laparoscopic approach. This method supports the vaginal cuff with paramesonephric remnants by second layer suturation.

## Materials and methods

**Figure 1:**
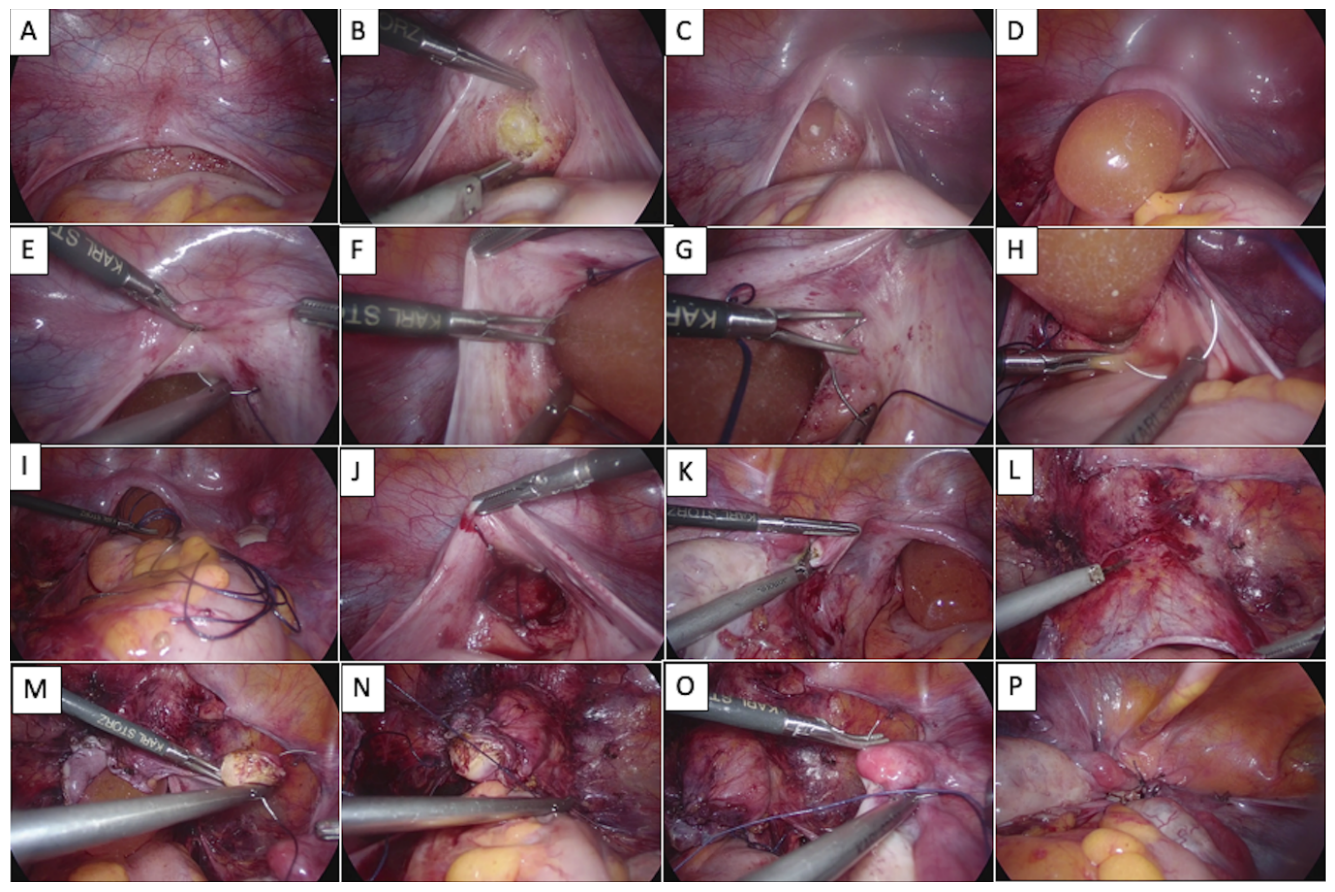
A-Laparoscopic view of rudimentary uterus, B-Peritoneal incision at top of the mold, C-Insertion of the thinner mold, D-Gradually dilatation with molds, E,F,G,H-Suturing the lateral edges of the peritoneum, I-Pulling down the sutures, J-View of the vaginal opening, K-Dividing the rudimentary horn into two parts, L-Sharp dissection of the peritoneum, M-Beginning the suturation from half of the rudimentary horn, N- Purse-string suturing of the first layer, O-Beginning the suturation from the other half of the rudimentary horn for the second layer, P-Purse string suturing othe second layer.

**Figure 2:**
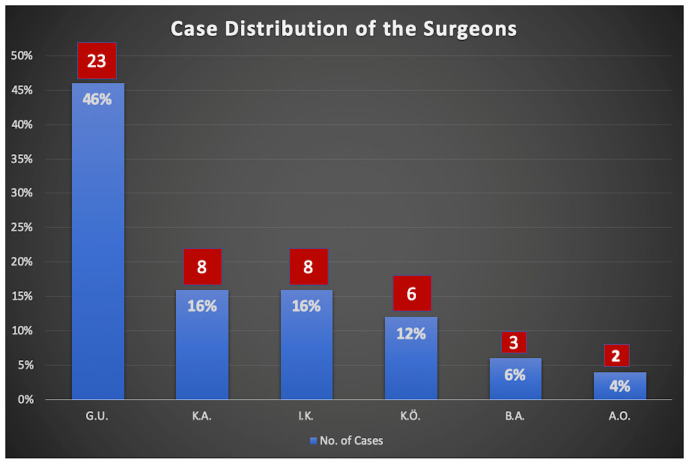
Case distribution depending on the surgeon.

This study was conducted at a tertiary university hospital. The ethical committee of the university approved the study protocol. Patients with MRKH syndrome who underwent Uncu-modified Davydov procedure between January 2008 and December 2021 were enrolled in the study. All patients underwent a detailed examination before the operation. Secondary sexual characteristics, chromosomal analysis, urinary system abnormalities, and detailed scanning for uterine remnants of the Mullerian ducts were evaluated. All patients were operated by one of six senior surgeons experienced in the procedure. The same technique described in our previous study was used in all operations (Uncu et al., 2018) (Figure 1). The surgeon-specific case distribution is presented in Figure 2.

### Postoperative Care

The patients were mobilized 24 hours after surgery. The surgeon applied the first mold exercise in order to teach the patient how to perform it. Patients were discharged on the third day after the operation. Patients were provided with a written guide for continued mold exercises and follow-up visits were arranged (Table III). The patient compliance, vaginal length & width, epithelization, and complications were noted at each visit. Sexual intercourse was advised as feasible three months post-op (provided there were no complications from coitus). The final vaginal length was measured one year after surgery.

**Table III:** 

Postoperative Day 1	The vaginal mold remains in place within the vagina through the day, allowing the patient to freely engage in activities such as walking, eating, and urinating. The initial mold exercise involving will be conducted by the surgeon.
Postoperative Day 2	The vaginal mold stays replaced in the vagina all day The patient will be educated how to perform the mold exercises The first self-exercise will be applied by the patient The patient will be discharged on postoperative Day-3
Postoperative First Week	The vaginal mold remains in place within the vagina through the night, allowing the patient to freely sleep in any position. The vaginal mold exercises will be performed three times a day. After each exercise, the mold will be reinserted into the vagina and kept in place for a duration of two hours.
Postoperative First Month	There is no need mold replacement during the nights The mold exercises will continue three times a day. Following each exercise, the duration the mold remains inside will be reduced to one hour.
Postoperative Second Month	The mold exercise will be conducted three times a day, with the mold being replaced after each session within a 30-minute timeframe. A follow-up appointment will be scheduled at the end of the second month.
Third Month Follow-Up	Patients will be asked about any complaints they may have, and their pain scores will be documented. A vaginal examination will be conducted, assessing factors such as vaginal length, vaginal epithelium metaplasia, and lubrication. If no issues are detected during the examination, patients will be advised to engage in sexual intercourse starting from the fourth month after the surgery

### Survey for Sexual outcomes

The electronic health records database was used to identify eligible patients. Patients were called and invited for the examination and FSFI survey completion. The Female Sexual Function Index (FSFI) is a validated and commonly used survey worldwide (Rosen et al., 2000). It is a questionnaire which measures sexual function in women. It contains nineteen questions. It was developed for the specific purpose of assessing domains of sexual functioning (e.g., sexual arousal, orgasm, satisfaction, pain) in clinical trials. The maximum point is 36 with the highest satisfaction, and the minimum is 2. Values below 26.55 are though to indicate female sexual dysfunction (Wiegel 2005). Oksuz and Malhan (2005) validated the FSFI in the Turkish population.

## Statistical Analysis

All the demographic data, surgical and survey outcomes were collected and transferred to a statistical package SPSS version 22.0 (Chicago, IL). Depending on the distribution, continuous variables are defined as mean +/- standard deviation (SD) or median (25th-75th percentile). Categorical variables are defined as percentages.

In order to conduct a comprehensive review of MRKH syndrome and surgical outcomes based on different approaches, we conducted a thorough search of PubMed using keywords such as neovagina, Mayer Rokitansky-Küster- Hauser Syndrome, FSFI (Female Sexual Function Index), Mullerian Agenesis, and laparoscopy. We specifically focused on identifying recent studies with relatively large sample sizes that reported both anatomical and sexual outcomes. The findings from these studies were compiled to create Table II for further analysis.

**Table II:** Summary of the sexual and anatomic outcomes of the novel published studies.

Author Year	Sample Size	Follow-Up	Methods	No. of Patients	Vaginal Length	Mean FSFI
Present Study	50	1 Year	Uncu Modification	50	8.4 ± 1.9	31.5 ± 3.9
Yang et al. 2022	53	2 Years	Vecchietti Davydov	32 21	8.3 ± 0.7 8.6 ± 1.2	27.2 ± 2.2 27.3 ± 3.5
Zhou et al. 2022	117	1 Year	McIndoe (SIS) Davydov	44 47	7.1 ± 0.8 8.1 ± 1.1	27.7 ± 3.5 28.1 ± 2.7
Rall et al. 2021	82	1 Year	Vecchietti	82	N/A	28.3 ± 5.3
Kang et al. 2020	45	1 Year	McIndoe / Davydov	28/17	8.1 ± 1.59	23.8 ± 3.6
Zhang et al. 2019	67	6 Months	McIndoe (SIS) McIndoe (Synthetic)	24 43	7.6 ± 0.8 7.5 ± 0.5	32.2 ± 2.2 31.6 ± 3.4
Sabatucci et al. 2019	39	1 Year	McIndoe (Autolog Mucosa)	39	8 (6-12)	27.2 (4.4-33.6)
Pastor et al. 2017	42	1 Year	Vecchietti	42	7 ± 9.6	29.9 ± 2.7
Zhang et al. 2017	40	1 Year	Mc Indoe (Synthetic)	40	>9	25.2 ± 7.7
Ding et al. 2015	75	1 Year	McIndoe (SIS) Davydov	34 41	6.8 ± 0.9 7.3 ± 1.3	25.6 ± 3.1 25.8 ± 2.8
Zhao et al. 2015	98	1 Year	Davydov Modified Davydov	36 62	8.6 ± 0.8 8.5 ± 1.2	29.5 ± 4.3 28.9 ± 4.6
Dong et al. 2015	28	1 Year	Vecchietti Davydov	13 15	8.8 ± 0.5 9.6 ± 0.5	26.8 ± 2 28.5 ± 1.7

*FSFI Score <26.55 is consistent with female sexual dysfunction.

## Results

Fifty women with MRKH syndrome underwent the Uncu-modified Davydov procedure between January 2008 and December 2021. The mean age was 27.3 + 4.7 years, and the mean body mass index was 23.4 + 3.6 kg/m2. Thirty-six of the patients had a normal urinary tract on imaging. Three patients were diagnosed with unilateral renal agenesis and three were diagnosed with double ureter.

There were four perioperative complications: three bladder injuries during the vaginal dissection through the Douglas pouch and one rectal serosa injury during peritoneal preparation. All of these injuries were intraoperatively and laparoscopically without recourse to further surgery.

There were four long-term complications: one vesicovaginal fistula, one rectovaginal fistula, and two vaginal stenoses (attributed to incompliance with regular mold exercise). The vesicovaginal fistula was successfully treated with Foley catheterization for two weeks. The rectovaginal fistula was diagnosed three months after surgery and required surgical repair and colostomy. The colostomy was successfully reversed six months thereafter.

**Figure 3:**
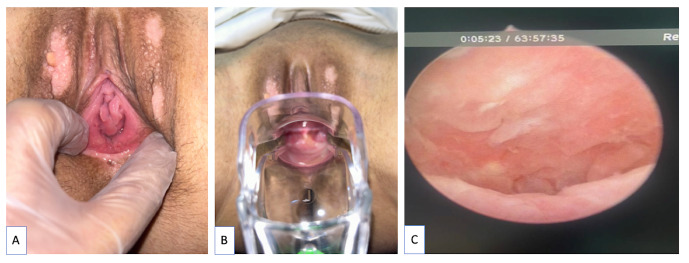
Long-Term Anatomic Outcomes; A- Healed vaginal orifice, B- The speculum can be easily inserted, C- The vaginal wall epithelium as seen during vaginoscopy.

The mean vaginal length at one-year control was 8.4 + 1.9 cm with enough width for sexual intercourse. A standard speculum could easily be inserted into the vagina of all but two patients. These two patients did not regularly exercise and dropped out from follow-up (Figure 3). The vaginal epithelium was completely formed at one- year control. There was no pelvic surgical stigmata on any patient.

There were thirty-six patients with a sexual partner who completed the FSFI survey. The mean FSFI score was 31.5 + 3.9 (minimum of 24, maximum of 36) (Table I).

**Table I:** Demographic parameters, Surgery and Survey Results.

Age (years) (SD)^a^		27.3 ± 4.7
Body Mass Index (kg/m2) (SD)		23.4 ± 3.6
Urinary Malformation		
	None	88% (44/50)
	Unilateral Renal Agenesis	6% (3/50)
	Double Ureter	6% (3/50)
Perioperative Complications		
	Bladder Injury	6% (3/50)
	Rectum Injury	2% (1/50)
Postoperative Complications		
	Vesico-vaginal fistula	2% (1/50)
	Recto-vaginal fistula	2% (1/50)
	Vaginal stenosis	4% (2/50)
Vaginal Length (cm) (Postoperative one-year) (SD)		8.4 ± 1.9
FSFI Score (SD)^b^		31.5 ± 3.9

a: Standard Deviation (SD); b: FSFI Score <26.55 is consistent with female sexual dysfunction.

## Discussion

In this study, the Uncu-modified Davydov procedure has been demonstrated to be a safe and effective treatment option with high female sexual function index scores for patients with MRKH syndrome. Since our first publication (Uncu et al., 2018), our unit has become a referral centre for this condition, and we are performing the operation more frequently.

There are two significant concerns for MRKH patients after diagnosis: the feasibility of sexual intercourse and the possibility of fertility. For the first, the solution is the creation of a functional vagina. The first-line therapy is the self-dilatation which requires patient compliance, regular exercises, and long-term patience (Willemsen and Kluivers, 2015; Gargollo et al., 2009; Patel et al., 2018). Considering the minimal occurrence of complications, such as urethral dilatation, and comparable sexual outcomes to surgical approaches (Cheikhelard et al., 2018), this method should be strongly recommended as the initial option for patients. The second-line therapy (surgical treatment) is an option when the non-surgical methods are ineffective or when patients request surgery. There are numerous surgical techniques, mainly in three subgroups; the McIndoe, Vecchietti, and Davydov procedures (Banister and McIndoe, 1938; Vecchietti, 1965; Davydov and Zhvitiashvili, 1974).

The Vecchietti technique is requires an abdominal approach, dissection of the vesicorectal septum and fixation of the vaginal “dilatation olive” using two sutures passing the vaginal stump, then externalizing the threads to a traction device through the abdominal wall (Vecchietti, 1965). This technique was initially developed as an open procedure and laparoscopic- or robotic-modified Vecchietti techniques have been reported (Cezar et al., 2014; Yang et al., 2021). The outcomes from the largest series of this procedure were published by Fedele et al. (2008) and Brucker et al. (2008). Fedele et al. (2008) described the sexual and anatomic outcomes of the 110 patients after Vecchietti procedure and reported a mean FSFI score of 29 + 3. Brucker et al. (2008) published the results of 101 patients and reported that mean vaginal length at six-months was 10.6 cm. Although good outcomes were reported, the traction device was required for approximately one week, this length of time may prove excessively painful for the patients.

The McIndoe technique is the first method developed to create a neovagina. This procedure involves the careful dissection of the space between the bladder & rectum and placing a stent covered with an autologous skin graft through the dissected space. Dilatation exercises must continue after surgery to avoid collapsing of the neovagina. Some novel developed methods like McIndoe use different materials instead of skin grafts. Small intestine mucosa, absorbable adhesion barrier, buccal mucosa, amnion membrane, peritoneum, tilapia fish skin and in-vitro cultured vaginal tissue are the known materials that have been used to create a neovagina (Teng et al., 2019; Sabatucci et al., 2019; Motta et al., 2017; Dias et al., 2020; Vatsa et al., 2017; Wu et al., 2020; Jackson and Rosenblatt, 1994; Rothman, 1972). This technique has a short recovery period as it does not involve any abdominal surgery. However, using graft materials is likely to increase the operative cost.

The last procedure is the Davydov operation. This entails pulling down the parietal peritoneum and suturing it to the vaginal introitus. This method has many advantages: firstly, a shorter recovery time than the Vecchietti procedure and a more cost-effective procedure than Vecchietti and McIndoe procedures. Using peritoneum eliminates the possible graft complications like hair formation (due to skin graft) heavy vaginal discharge (due to intestinal graft), foreign body reaction, and granulation for synthetic grafts.

Our Uncu-modified Davydov procedure seems to provide both a robust vaginal dome with remnant supported double-layer saturation and an adequate average vaginal length at one-year follow-up. The majority of studies consider a vaginal length of 6 cm to be sufficient. In addition to achieving a suitable vaginal length, our patients also demonstrated favourable outcomes in terms of their FSFI scores (mean FSFI: 31.4 + 3.9) when compared to patients who underwent surgical procedures such as Davydov, Vecchietti, or McIndoe techniques. (Zhao et al., 2015; Dong et al., 2015; Yang et al., 2022; Zhou et al., 2022; Rall et al., 2021; Kang et al., 2020; Zhang et al., 2019; Pastor et al., 2017; Zhang et al., 2017; Ding et al., 2015; Zhao et al., 2015) (Table II).

Although the present study recommends that this surgical approach is satisfactory with long-term sexual and anatomic outcomes, it should not be forgotten that the first-line treatment option remains self-dilatation. Many of our patients declined self- dilatation due to either their cultural or religious prohibition of premarital sexual activity.

There are some concerns with our surgical approach. First, the complication rates seem relatively high yet similar to other surgical methods (Cheikhelard et al., 2018). Four perioperative complications (8%) were repaired during intraoperatively without sequelae. When we retrospectively analysed these complications, we found that each surgeon (other than Prof. Uncu) had one injury, and each injury occurred during the respective surgeon’s learning phase with regard to this procedure. More recently, there have been no perioperative or postoperative complications for the last 30 cases. Within these last 30 cases, there have been two vaginal stenoses (4%), primarily thought to be due to noncompliance. We have developed a postoperative follow-up chart for these which details how to exercise with the vaginal mold in terms of frequencies, duration, techniques, when to attempt intercourse and follow-up times) (Table II).

**Figure 4:**
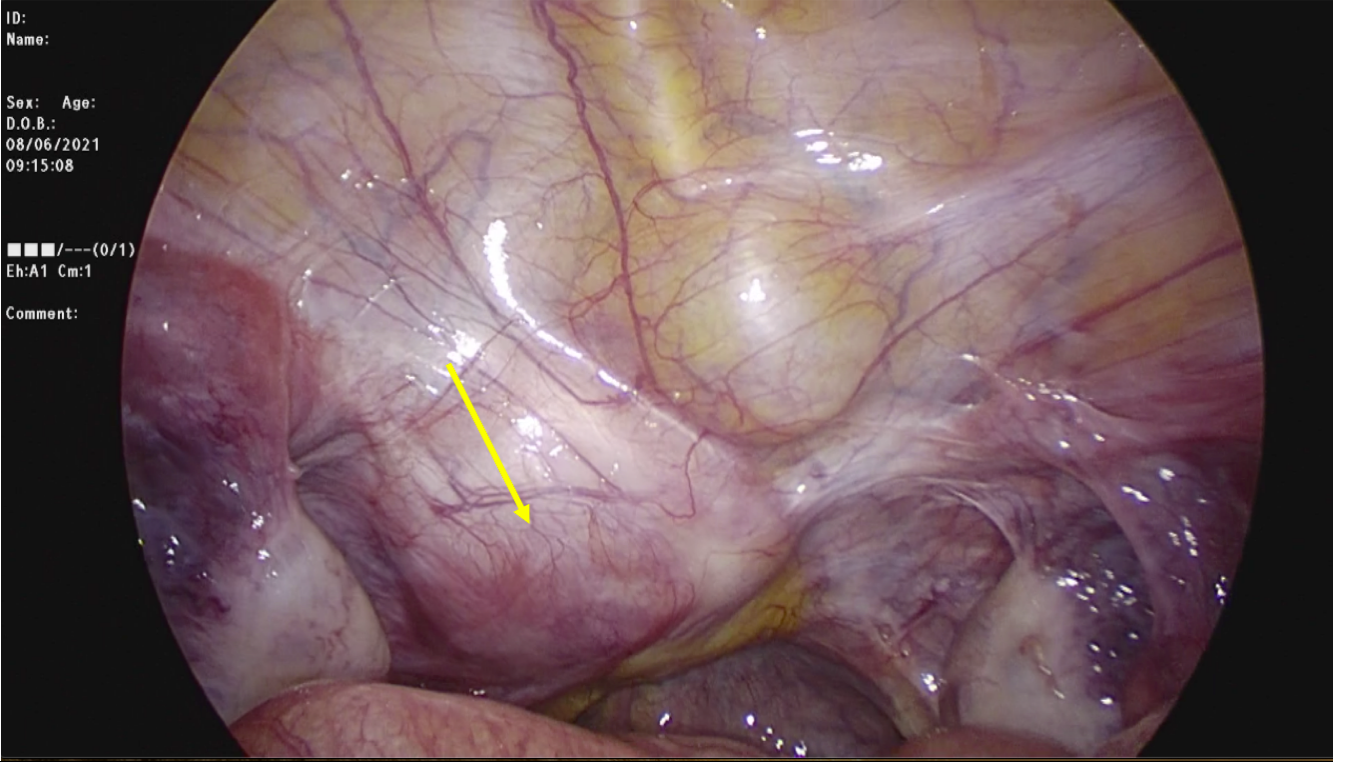
The laparoscopic view of the pelvis (after seven years of neovagina creation) - the yellow arrow indicates the fibroid on the neovaginal cuff.

Another major concern for women who have had this operation is the prospect of motherhood. We performed a phone survey to assess their ambitions in this regard. There were three options: uterine transplantation, maternal surrogacy, and adoption. Almost 70% preferred uterine transplantation. The low rate of surrogacy was thought mainly to be due to the prohibition of surrogacy by the government because of religious beliefs. Although the most preferred option was uterine transplantation, no patient has yet to undergo this operation. This may be due to our country’s low frequency of uterine transplantation. As such, we have yet to observe the feasibility of uterine transplantation after this procedure. Nevertheless, we would expect our procedure would not make uterovaginal anastomosis difficult as we had the chance to perform a ‘second look’ laparoscopy. The patient had new-onset dyspareunia seven years after neovagina creation and was found to have a fibroid on the neovaginal cuff. We performed a laparoscopic myomectomy. There were no adhesions that might complicate the possible uterine transplantation (Figure 4).

Our study has some strengths and limitations. The strengths are the relatively large number of patients undergoing the same procedure (as MRKH syndrome is a rare condition) and the use of a globally-validated sexual function survey (FSFI) are the strengths of our study. The absence of a control group with another surgical approach limits the interpretation of our results.

In conclusion, the Uncu-modified Davydov procedure is a satisfactory surgical approach with high female sexual function index scores for Mayer-Rokitansky-Küster-Hauser Syndrome patients. Nevertheless, it is important to note that the self-dilatation method is the safest first-line option as it minimizes the risk of complications. Patients who have not achieved success with the primary treatment of self-dilatation should be informed about the potential complications and the need for post-operative mold exercises that accompany a surgical approach.
